# Protocol for systematic review: patient decision aids for total hip and knee arthroplasty decision-making

**DOI:** 10.1186/s13643-020-01549-6

**Published:** 2021-01-04

**Authors:** Lissa Pacheco-Brousseau, Marylène Charette, Dawn Stacey, Stéphane Poitras

**Affiliations:** 1 School of Rehabilitation Sciences, Faculty of Health Sciences, University of Ottawa, 451 Smyth Road, Ottawa, Ontario Canada; 2 Population Health, Interdisciplinary School of Health Sciences, Faculty of Health Sciences, University of Ottawa, Ottawa, Ontario Canada; 3 School of Nursing, Faculty of Health Sciences, University of Ottawa, Ottawa, Ontario Canada; 4 Clinical Epidemiology Program, The Ottawa Hospital Research Institute, Ottawa, Ontario Canada

**Keywords:** Patient decision aids, Total joint arthroplasty, Total hip arthroplasty, Total knee arthroplasty, Shared decision-making

## Abstract

**Background:**

Total hip and knee arthroplasty are a highly performed surgery; however, patient satisfaction with surgery results and patient involvement in the decision-making process remains low. Patient decision aids (PtDAs) are tools used in clinical practices to facilitate active patient involvement in healthcare decision-making. Nonetheless, PtDA effects have not been systematically evaluated for hip and knee total joint arthroplasty (TJA) decision-making. The aim of this systematic review is to determine the effect of patient decision aids compared to alternative of care on quality and process of decision-making when provided to adults with hip and knee osteoarthritis considering primary elective TJA.

**Methods:**

This systematic review will follow the Cochrane Handbook for Systematic Reviews. This protocol was reported based on the PRISMA-P checklist guidelines. Studies will be searched in CINAHL, MEDLINE, Embase, PsycINFO, and Web of Science. Eligible studies will be randomized control trial (RCT) evaluating the effect of PtDA on TJA decision-making. Descriptive and meta-analysis of outcomes will include decision quality (knowledge and values-based choice), decisional conflict, patient involvement, decision-making process satisfaction, actual decision made, health outcomes, and harm(s). Risk of bias will be evaluated with Cochrane’s risk of bias tool for RCTs. Quality and strength of recommendations will be appraised with Grades of Recommendation, Assessment, Development and Evaluation (GRADE).

**Discussion:**

This review will provide a summary of RCT findings on PtDA effect on decision-making quality and process of adults with knee and hip osteoarthritis considering primary elective TJA. Further, it will provide evidence comparing different types of PtDA used for TJA decision-making. This review is expected to inform further research on joint replacement decision-making quality and processes and on ways PtDAs facilitate shared decision-making for orthopedic surgery.

**Systematic review registration:**

PROSPERO CRD42020171334

## Introduction

Osteoarthritis (OA) is a leading cause of disability worldwide impacting health on an individual and population level [1, 2]. OA of the hip and knee is a highly prevalent disease in Canada affecting 38 to 45% of Canadians aged 50 years and older [3]. Clinical guidelines for the management of OA recommend arthritis education, structured land-based exercise programs, and pharmacologic treatment [4, 5]. When conservative treatment fails to alleviate symptoms, total joint arthroplasty (TJA) is the recommended treatment [6, 7]. Nonetheless, TJA requires trading off benefits and risks as it is one treatment option amongst many others with no single “best” choice [8]. TJA is an elective surgery and the third most performed inpatient surgery in Canada, with 58,492 total hip arthroplasty (THA) and 70,502 total knee arthroplasty (TKA) performed in 2018 [9]. This represents a volume increase of 17.0% compared to 2013 [9]. Similar trends are seen worldwide and numbers are expected to continue rising [10–13]. This phenomenon is concerning for healthcare systems considering the inpatient and outpatient resources needed for TJA procedure [9, 14, 15].

OA represents 80% of the reason for undergoing TJA and the majority of recipients are 65 years and older [9, 13, 16]. TJA has been shown to successfully reduce pain and improve function, mobility, and health-related quality of life for patients with severe OA [17–20]. Concurrently, there are potential harms associated with this surgery such as joint infection, venous thrombosis, cardiovascular complications, post-operative pain and stiffness, and life-threatening complications [21–23].

Twenty to 45% of joint arthroplasty are considered of questionable appropriateness [8], and 10 to 30% of patients are not satisfied with the outcomes [24–27]. The first factor contributing to these statistics is the limited evidence-based criteria for TJA appropriateness [8]. Therefore, healthcare professionals and surgeons use their expert opinion in the decision-making process. However, studies have shown discrepancies between healthcare professional opinion and patient preferences [8, 28–34]. Additionally, many studies have shown insufficient information provided to patients [35, 36] and most patients have unrealistic outcome expectations [37, 38].

Involvement of patients in their healthcare decision is well documented to improve patient satisfaction, sense of control, health outcomes, treatment quality, and adherence, and reduce healthcare costs [39–44]. Shared decision-making (SDM) is a process actively including patients as partners in making healthcare decisions together with their clinician(s) and involves providing them evidence-based knowledge on options and discussion of their values and preferences [45–47]. A recent systematic review has demonstrated the effectiveness of SDM for elective surgery to improve decision quality and decrease decisional conflict [48].

To facilitate SDM, patient decision aids (PtDAs) are tools which can take many forms (pamphlets, videos, Internet-based) that can be used in the clinical encounter or in preparation for the clinical encounter. They support SDM by making the decision explicit and providing information about benefits, harms, and uncertainty of options. Furthermore, they also guide patients in values clarification to achieve agreement between features of the option chosen and personal values [49, 50]. Contrary to educational materials, PtDAs focuse on making explicit the decision, options, and associated outcomes and provides guidance on clarifying personal values [49]. A Cochrane review including 105 studies and 31,043 individuals demonstrated that PtDAs improve decision quality (increasing patient knowledge, fostering more accurate expectations and risk perceptions, supporting clarification of what is important to patients, and helping patients choose the best option associated to their values) and decision-making process (promoting clinician-patient decision discussion, increasing patient participation in the decision-making process, decreasing decisional conflict, and promoting a positive healthcare experience) [49]. Moreover, it was demonstrated that PtDAs used in the context of elective surgery, patients are less likely to choose surgery when they understand alternative options [48, 49, 51]. Further, PtDAs were shown to hold similar long-term cost and surgery rates compared to usual care [51], short-term cost saving [52], but unclear long-term cost-effective properties [52].

A recent systematic review exploring features and impact of SDM/PtDA interventions for knee arthritis TJA decision-making found that most trials did not include all key aspects of SDM [53]. The authors recognized the need for future research to focus on assessing the effect of PtDAs and SDM on treatment decision, functional outcomes, and satisfaction [53].

With the proportion of seniors living in industrialized countries growing rapidly [54, 55], it is fundamental to improve current TJA decision-making processes that can lead to improved healthcare efficiency, better patient expectations, better patient and surgeon experiences, and reduced burden on the healthcare system. To date, no systematic review has focused on assessing the effect of PtDAs for adults with OA considering primary elective THA and TKA.

## Objectives

The aim of this systematic review is to determine the effect of patient decision aids compared to alternative of care on quality and process of decision-making when provided to adults with hip and knee osteoarthritis considering primary elective TJA. Specifically, objectives related to the quality of the decision and decision-making process are:

1. To evaluate the effect of PtDA on increasing patient decision quality including knowledge, and values-based choice for primary elective TJA decision-making
2. To evaluate the effect of PtDA on reducing decisional conflict and increasing patient participation for primary elective TJA decision-making
3. To compare the effect of different PtDAs on decision quality, decisional conflict, and patient participation

Objectives related to decision-making outcomes are:

4) To evaluate the effect of PtDA on increasing decisional satisfaction for primary elective TJA decision-making
5) To evaluate the effect of PtDA on the actual decision and health status for primary elective TJA decision-making
6) To evaluate the effect of PtDA on reducing harm for primary elective TJA decision-making

## Methods

This protocol was guided by the Cochrane Handbook for Systematic Reviewer [56]. This protocol was reported based on the Preferred Reporting Items for Systematic Review and Meta-analysis Protocols (PRISMA-P) checklist guidelines [57].

### Eligibility criteria

See Table 1 for the inclusion and exclusion criteria.

**Table 1:** Inclusion and exclusion criteria

PICO framework	Inclusion	Exclusion
Population	• Adults aged 18 years and older • Hip/knee OA • Primary elective THA/TKA	TJA for: • Trauma • Emergency • Previous TJA • Revision • Prosthesis replacement • Other conditions than OA
Intervention	• PtDA for TJA • PtDA needs to meet the six minimum definition IPDAS criteria • PtDA of any types • PtDA exposition: before, during, and after consultations	• Other SDM strategies • Not enough information to evaluate PtDA content
Comparators	• Alternative of care (another PtDA) • Standard of care • No intervention	
Outcomes	Primary outcomes: • Decision quality (knowledge, values-based choice) • Decision-making process (decisional conflict, patient participation) Secondary outcomes: • Decision-making results (decisional satisfaction, actual decision made, health status, and harm)	• Do not report any of the outcomes of interest
Study design	• RCT	• All other designs, commentaries, dissertations, and conference abstracts
Languages	• All languages that can be translated	

#### Population

Eligible studies will involve adult patients considering primary elective THA and TKA as treatment for OA since this health problem is the primary reason for TJA [9, 13, 16]. Studies will be excluded if involving patients considering primary TJA secondary to trauma, emergency, revision, prosthesis replacement, and any other condition except for OA.

#### Intervention

The intervention of interest is the use of any PtDA for patients suffering from OA having to make the decision to undergo primary TJA in any settings. As defined per the International Patient Decision Aids Standards (IPDAS) Collaboration [58], PtDAs are evidence-based tools developed to help patients make a decision while considering two or more healthcare options (including status quo). They are intended to provide information on options and help patients clarify their values associated with the different options. Decision aids are tools to supplement healthcare professional consultations [49, 58]. According to IPDAS [58], minimum criteria to be defined as a PtDA are (1) to make explicit that a decision has to be made, (2) helping patients to choose amongst options, (3) presentation of associated positive and negative outcome for each option, (4) outcomes presented are relevant to health status, (5) the tool should not favor any option, and (6) the tool needs to assist in personal values clarification. To ensure the quality of PtDAs, studies not providing information pertaining to the preceding criteria will be excluded if the corresponding author cannot provide the required information. Eligible studies will involve PtDA of any type (videos, pamphlets, Internet-based). Furthermore, interventions with patients exposed to a PtDA either before, during, or after consultations will be eligible since PtDAs support can be used in any of these time points [49]. This systematic review will not focus on other SDM strategies.

#### Comparators

Eligible studies will involve patients exposed to a PtDA compared to patients following alternative care, standard of care, or no intervention. This systematic review will consider studies comparing two different types of PtDA. TJA decision-making standard of care includes the use of general educational material [44] which provide condition information and associated usual treatment. Educational materials provide broad perspective, are not focus on decision elements, and do not actively prepare patients to be involved in the decision-making [49, 59]. Studies will be excluded if they compared PtDA with another SDM strategy (decision coaching).

#### Outcomes

Based on IPDAS criteria [58], primary outcomes related to the decision quality will be (a) knowledge assessed with Decision Quality Instrument (DQI)-Hip and Knee Osteoarthritis [60], or any other; and (b) values-based choice with patient reporting or any instrument. Primary outcomes related to the decision-making process will be (a) decisional conflict (defined as the uncertainty of choosing options) assessed with the SURE scale [61], Decisional Conflict Scale (DCS) [62], Decision Quality Instrument (DQI)-Hip and Knee Osteoarthritis [60], or any other; and (b) patient participation assessed with patient reporting or any other instrument. Secondary outcomes related to decision-making results will be (a) decisional satisfaction assessed with the Decision Attitude Scale [63] or any other, (b) actual decision made assessed with any scale, (c) health status (including quality of life and emotional health) evaluated with the EQ-5D [64] or any other, and (d) harm (including emotional distress) measured with the Decision Regret Scale [65], Kessler Psychological Distress Scale [66], or any other. To be eligible for inclusion, at least one of the outcomes must be reported.

#### Study types and others

For quality purposes, this systematic review will only include randomized control trials (RCTs). Eligible studies will be in all languages that can be translated and published before study inception.

### Data sources and search strategy

This systematic review will include studies searched in the following electronic databases: CINAHL, MEDLINE, Embase, PsycINFO, and Web of Science. The search strategy was created by the first author (LPB) with the help of an expert medical librarian (MCD) using keywords and Medical Subject Headings (MeSH) terms related to THA, TKA, TJA, OA, PtDA, and SDM (see the Appendix for the MEDLINE full search strategy). The search strategy will be compared to the PRESS guidelines and modified accordingly [67]. The search strategy will be adapted to the other databases with the help of an expert medical librarian. Reference lists of included studies will also be manually screened to ensure all eligible studies have been included by two independent reviewers (LPB and MC).

### Study records

#### Data management and selection process

All search results will be imported to Zotero reference management software [68] and Covidence web-based platform for systematic review [69]. After duplicate removal, study selection will be conducted by two independent reviewers (LPB and MC) in two phases using study eligibility criteria. The first phase will consist of screening titles and abstracts for potential eligible studies. All studies appearing eligible or unsure will be passed to the next phase. The second phase will consist of screening the full text of eligible studies. When full text is not available, corresponding authors will be contacted. Both reviewers will record the selection process in Covidence as well as associated justification for study exclusion. Divergences between reviewers will be discussed until consensus. A third reviewer (SP) will adjudge if no consensus is obtainable. Study selection will be illustrated in the final manuscript using the PRISMA flow diagram.

#### Data extraction

Data extraction will be conducted independently by LPB and MC using a predeveloped data extraction Excel form. To ensure adequacy of extraction, LPB and MC will pilot one study together. Disagreements will be solved by discussion or if necessary with a third reviewer (SP). Data extraction will include (1) general publication information such as the year of publication, journal, country, author list, funding, and conflict of interest; (2) study details such as design, sample size, and setting; (3) study population information such as demographic characteristics and characteristic of the decision to be made (THA or TKA); (4) PtDA information such as PtDA name, type, and IPDAS criteria; (5) intervention details such as patient exposure to PtDA and time points; (6) control group alternative or standard of care details; (7) outcome measures prespecified with timeframe; and (8) result summary including effect size and dropout rates. If available in articles, THA and TKA results will be extracted and reported separately. Corresponding authors will be contacted for further information if deemed necessary.

### Risk of bias

Methodological quality of all included studies will be appraised independently by two reviewers (LPB and MC) with the Cochrane’s risk of bias tool. Accordingly, risk of bias assessment will include selection bias, performance bias, detection bias, attrition bias, reporting bias, and other biases [70]. Based on the tool’s criteria, each item will be classified as high, low, or unclear risk. Justifications will be documented in a separate file with associate quotes from articles. Disagreements between reviewers will be resolved with discussion or if necessary by a third party (SP). Risk of bias for all included studies will be illustrated in a risk of bias figure in the final manuscript.

### Data synthesis

The results of this systematic review will be descriptively synthesized. The synthesis will include basic characteristics of included studies and outcome measured. Subsequently, primary and secondary outcomes and comparison between PtDA and comparator will be synthesized. Studies will be subdivided into two comparison categories: studies comparing PtDA with standard of care and studies comparing two PtDAs. Consequently, the synthesis will be twofold: (1) the effect of PtDA on TJA decision-making and (2) comparing the effect of different PtDA. Each category will be subdivided by demographics (age, sex, ethnicity, and socioeconomic status), past-medical history of arthroplasty, and TKA vs THA since these have been shown to have an impact on TJA decision-making [71–74]. Risk of bias will be taken into consideration for the synthesis and will be summarized narratively.

A meta-analysis will be considered once data collection is completed and will depend on the number of included studies, their risk of bias, and their clinical, methodological, and statistical homogeneity. If outcomes were measured using the same or a similar instrument, the meta-analysis will consist of pooling results of categories using the random effects model. Results will be illustrated in a forest plot. Dichotomous measures will be analyzed by the number of events in the alternative or standard of care group compared to the intervention group. Risk ratio with 95% confidence interval (CI) will be reported. Continuous measures will be analyzed with a comparison of intervention versus alternative or standard of care group means. Standard deviation (SD) with 95% CI will be reported. Heterogeneity of studies will be visually investigated and calculated with the *I*^2^ statistic. *I*^2^ of 0–40% will be considered of low heterogeneity, 30–60% moderate heterogeneity, 50–90% substantial heterogeneity, and 75–100% considerable heterogeneity [56]. If considerable heterogeneity is found, subgroup analyses will be performed such as patient sex and age, TKA and THA [71–74], patient history of TJA [74], and risk of bias score. Publication bias will be evaluated with funnel plots if the systematic review includes more than 10 studies [75]. All statistics will be performed using RevMan Web [76]. The descriptive synthesis and statistical analyses will be performed by the first author (LPB).

### Confidence in cumulative evidence

The quality and strength of recommendations of the narrative and meta-analysis synthesis will be appraised through the Grades of Recommendation, Assessment, Development and Evaluation (GRADE) by two independent authors (LPB and MC) [56]. A “Summary of findings” table will be created providing assumed risk with associated source and rationale for each outcome by two reviewers (LPB and MC). The certainty of evidence will be rank as high, moderate, low, or very low based on (1) risk of bias, (2) inconsistency, (3) indirectness, (4) imprecision, and (5) publication bias [56, 77]. The online GRADEpro GDT tool will be used [78].

## Discussion

To our knowledge, no systematic review has been conducted on PtDAs for adults with OA considering primary elective THA and TKA decision-making. This review will complement the systematic review of Riddle et al. [53] by focusing on PtDAs by (1) providing a summary of RCTs findings on PtDAs effect on decision-making quality and process of adults with knee and hip OA considering primary elective TJA and by (2) providing evidence comparing different types of PtDA used for hip and knee TJA decision-making.

With the increasing senior population leading to increase TJA demands [9, 54, 55], it is fundamental to improve current TJA decision-making processes that can lead to improved healthcare efficiency and better patient expectations. Further, SDM is consistent with patient-centered care, defined as “providing care that is respectful of and responsive to individual patient preferences, needs and values and ensuring that patient values guide all clinical decisions,” which is a national goal for medical practices [79, 80]. Findings from this systematic review will inform further research on joint replacement decision-making quality and processes and on ways PtDAs facilitate SDM for orthopedic surgery.

Strength of this review is the a priori rigorous study methods including selection criteria with associated justification, exhaustive literature search strategy, and study selection, data extraction, and appraisal by two independent reviewers. Rigorous protocol is fundamental for conducting a good-quality systematic review. The limitation of this review is solely focusing on RCTs evaluating the effect of PtDA compared to standard of care or another PtDA for quality purpose. This may lead to the exclusion of good-quality observational studies. Further, this review will only include studies with adults considering primary elective TJA; therefore, findings will not be generalizable to adults considering TJA for any other reasons.

## Data Availability

Not applicable.

## Appendix

MEDLINE full search strategy

1. Arthroplasty, Replacement, Hip/

2. Arthroplasty, Replacement, Knee/

3. Hip Prosthesis/

4. Knee Prosthesis/

5. hip$ arthroplast*.ti,ab.

6. hip$ replacement$.ti,ab.

7. (total adj3 hip$ adj3 (replacement$ or arthroplast*)).ti,ab.

8. (hip$ adj3 prosthe*).ti,ab.

9. hip$ prosthe*.ti,ab.

10. knee arthroplast*.ti,ab.

11. knee replacement$.ti,ab.

12. (total adj3 knee adj3 (replacement$ or arthroplast*)).ti,ab.

13. knee prosthe*.ti,ab.

14. (total adj3 joint$ adj3 (replacement$ or arthroplast*)).ti,ab.

15. joint$ replacement$.ti,ab.

16. joint$ arthroplast*.ti,ab.

17. 1 or 2 or 3 or 4 or 5 or 6 or 7 or 8 or 9 or 10 or 11 or 12 or 13 or 14 or 15 or 16

18. exp decision making/ or choice behavior/

19. decision support techniques/

20. Patient Participation/

21. Patient Education as Topic/

22. Decision Support Systems, Clinical/

23. Decision Trees/

24. exp Decision Making, Computer-Assisted/

25. (decision making or choice behavior).mp. and informed consent.sh.

26. patient$ decision aid$.ti,ab.

27. (patient$ adj3 (decision$ or aid$)).ti,ab.

28. ((decision* or decid*) adj3 (support* or aid* or tool* or instrument* or technolog* or technique* or system* or program* or algorithm* or process* or method* or intervention* or material*)).ti,ab.

29. (decision adj3 (board* or guide* or counsel*)).ti,ab.

30. ((risk communication or risk assessment or risk information) adj3 (tool* or method*)).ti,ab.

31. interactive health communication*.ti,ab.

32. (interactive adj (internet or online or graphic* or booklet*)).ti,ab.

33. (interacti* adj3 tool*).ti,ab.

34. ((interactiv* or evidence based) adj3 (risk information or risk communication or risk presentation or risk graphic*)).ti,ab.

35. shared decision making.ti,ab.

36. (computer* adj3 decision making).ti,ab.

37. (informed adj3 (choice* or decision*)).ti,ab.

38. adaptive conjoint analys#s.tw.

39. 18 or 19 or 20 or 21 or 22 or 23 or 24 or 25 or 26 or 27 or 28 or 29 or 30 or 31 or 32 or 33 or 34 or 35 or 36 or 37 or 38

40. exp Randomized Controlled Trials as Topic/

41. research design/ or control groups/ or double-blind method/ or random allocation/

42. exp controlled clinical trial/

43. single-blind method/

44. randomi*.ti,ab.

45. (randomi* adj3 (control* or trial$)).ti,ab.

46. randomly.ti,ab.

47. trial.ti,ab.

48. placebo.ti,ab.

49. 40 or 41 or 42 or 43 or 44 or 45 or 46 or 47 or 48

50. 17 and 39 and 49

## Abbreviations

CI: Confidence interval
DCS: Decisional Conflict Scale
DQI-H/K: Decision Quality Instrument-Hip or Knee
GRADE: Grades of Recommendation, Assessment, Development and Evaluation
IPDAS: International Patient Decision Aids Standards
MeSH: Medical Subject Headings
OA: Osteoarthritis
PtDAs: Patient decision aids
RCT: Randomized control trial
SD: Standard deviation
SDM: Shared decision-making
THA: Total hip arthroplasty
TJA: Total joint arthroplasty
TKA: Total knee arthroplasty
